# Antifungal and antibiofilm activities of chromones against nine *Candida* species

**DOI:** 10.1128/spectrum.01737-23

**Published:** 2023-10-24

**Authors:** Jin-Hyung Lee, Yong-Guy Kim, Yeseul Kim, Jintae Lee

**Affiliations:** 1 School of Chemical Engineering, Yeungnam University, Gyeongsan, South Korea; University of Debrecen, Debrecen, Hungary

**Keywords:** biofilm, *Candida*, chromone, chromone-3-carbonitrile, hyphae

## Abstract

*Candida* species are the most common cause of fungal infections, and thus the emergence of antifungal-resistant species and biofilm formation by *Candida* further threatens the limited availability of antifungal agents. We investigated the antifungal and antibiofilm activities of 27 chromones against nine *Candida* species, including *Candida albicans, Candida glabrata*, *Candida parapsilosis*, and *Candida auris*. Four chromone-3-carbonitriles exhibited good antifungal activity with minimum inhibitory concentrations from 5 to 50 µg/mL and significantly inhibited biofilm formation by *C. albicans*. Furthermore, these four chromone-3-carbonitriles markedly inhibited cell aggregation and hypha formation, which are virulence factors and prerequisites for *C. albicans* biofilm formation. Transcriptomic analyses showed that the most active 6-bromochromone-3-carbonitrile downregulated the expressions of hypha-forming and biofilm-related genes (*TEC1* and *UME6*) but upregulated the hyphal regulator *UCF1*. Toxicity assays conducted using plant and nematode models showed that the four chromone-3-carbonitriles were non-to-mildly toxic. This study suggests that chromones bearing the 3-carbonitrile motif should be considered potential antifungal agents against *Candida* species.

**IMPORTANCE:**

The persistence of *Candida* infections is due to its ability to form biofilms that enable it to resist antifungals and host immune systems. Hence, inhibitions of the biofilm formation and virulence characteristics of *Candida* sp. provide potential means of addressing these infections. Among various chromone derivatives tested, four chromone-3-carbonitriles showed antifungal, antibiofilm, and antivirulence activities against several *Candida* species. Their mode of action has been partially revealed, and their toxicity is reported here using nematode and plant models.

## INTRODUCTION

*Candida* species are the common cause of invasive mycotic diseases, and some of these opportunistic pathogens, such as *Candida albicans, Candida glabrata*, *Candida parapsilosis*, and *Candida auris,* can cause life-threatening infections in immunocompromised individuals. Antifungal drugs, such as polyenes, azoles, and echinocandins, are used to treat invasive mycoses, but the emergence of drug-resistant *Candida* species has limited the effectiveness of the traditional antifungal armamentarium (1, 2).

*Candida* species have several virulence factors, such as hypha and biofilm formation, proteases, phospholipases, and lipases. In particular, biofilm-forming *C. albicans*, *C. glabrata*, *C. parapsilosis*, and *C. tropicalis* isolates have been associated with significantly higher mortality rates (3). *Candida* species adhere to host tissues and indwelling medical devices, such as catheters and implants, and then form drug-resistant biofilms, which enable them to withstand host immune defense systems and antifungal drugs. Furthermore, morphogenetic conversion (the ability to reversibly switch between yeast and filamentous hyphal forms) plays a pivotal role in the biofilm development and pathogenicity of *C. albicans* because the hyphal form can invade epithelial cells and cause tissue damage (4).

Chromones are oxygen-containing benzoannelated γ-pyrone heterocycles and are widely distributed in plants. Chromone scaffolds are privileged due to their diverse pharmacological properties, which include antiallergic, anti-inflammatory, antidiabetic, antitumor, antimicrobial activities, their synthetic accessibilities, and structural diversities (5, 6). Although the chromone backbone has no or little effect on *Candida* strains, several chromone derivatives, such as sulfonamide-derived chromones (7), hydroxy-pyrazolyl chromones (8), azo chromophores (7), oxepinochromones (9), methoxy-fluorophenyl chromones (10), cyclohexyl-substituted chromones (11), and chromanone A (12), have been shown to possess antifungal activities against some *Candida* strains.

The present study was undertaken to investigate the antifungal and antibiofilm activities of various chromone derivatives, including halogenated-, methyl-, formyl-, carboxyl-, and carbonyl-chromones, against nine *Candida* species, and to investigate the mechanisms responsible. Initially, we screened 27 commercially available chromone derivatives for antifungal activity against *C. albicans*, *C. glabrata*, *C. parapsilosis*, and *C. auris* and then investigated the effects of the four (6-bromochromone-3-carbonitrile, chromone-3-carbonitrile, 6-isopropylchromone-3-carbonitrile, and 6-methylchromone-3-carbonitrile) that most inhibited biofilm and hypha formation. Scanning electron microscopy (SEM) and live imaging microscopy were used to observe cell morphologies and phenotypic switching, and quantitative real-time reverse transcription PCR (qRT-PCR) was used to examine the molecular bases of their activities. Structure-activity relationships and absorption, distribution, metabolism, and excretion (ADME) simulation were performed, and nematode and plant models were used to explore compound toxicities.

## RESULTS

### Antifungal activities of chromones against *Candida* species

The antifungal activities of the 27 chromones were initially determined using minimum inhibitory concentration (MIC) assays against nine *Candida* strains, viz., two *C. albicans*, two *C. glabrata*, three *C. parapsilosis*, including a clinical isolate of *Candida parapsilosis* ATCC 22019 and two *C. auris* strains. The standard anti-*Candida* amphotericin B was used as a positive control and all nine strains were sensitive to amphotericin (MIC < 5 µg/mL). The antifungal efficacy of 27 chromones’ patterns differed between genera but was similar between species (Table 1). Two *C. albicans* strains, including a fluconazole-resistant *C. albicans* DAY185 strain (fluconazole MIC > 1,024 µg/mL), were most sensitive to chromones, whereas two *C. auris* strains (KCTC 17809 and 17810) were least sensitive except 3-bromo-6-chlorochromone with an MIC of 20 µg/mL (71.7 µM). Notably, four chromone-3-carbonitriles, namely, 6-bromochromone-3-carbonitrile (**6** in Table 1), chromone-3-carbonitrile (**12**), 6-isopropylchromone-3-carbonitrile (**23**), and 6-methylchromone-3-carbonitrile (**25**) exhibited good antifungal activity with MICs in the range of 5–50 µg/mL against *C. glabrata*, *C. parapsilosis,* and *C. albicans* species. It appears that all four chromone-3-carbonitriles were fungicidal since the treatment of any chromone-3-carbonitriles at 20 µg/mL (two or four times of their MICs) for 24 h killed all *C. albicans* cells (no CFU detected). Additional CFU assay was performed with 6-bromochromone-3-carbonitrile at 1× MIC (5 µg/mL) and 2× MIC (10 µg/mL). It appears that the compound at 1× MIC (5 µg/mL) completely killed *C. albicans* after 2 h contact as no CFU was detected (Fig. S1). Furthermore, the antifungal activity of these chromones was similar in four clinical isolates of *C. albicans* and *C. tropicalis* (Table S1).

**TABLE 1:** MICs of the 27 chromones studied^*a*^

#	Name	MIC (µg/mL)									
		*C. albicans* DAY185	*C. albicans* ATCC 10231	*C. glabrata* ATCC 2001	*C. glabrata* KCCM 50701	*C. parapsilosis* ATCC 7330	*C. parapsilosis* KCCM 50030	*C. parapsilosis* ATCC 22019	*C. auris* KCTC 17809	*C. auris* KCTC 17810
1	2-Amino-3-formylchromone	>100	>100	>100	>100	>100	>100	>100	>100	>100
2	2-Amino-6-chloro-3-formylchromone	>100	>100	>100	>100	>100	>100	>100	>100	>100
3	3-Bromochromone	>100	>100	>100	>100	100	100	100	100	100
4	6-Bromo-4H-chromen-4-one	>100	>100	>100	>100	>100	>100	>100	>100	>100
5	6-Bromochromone-2-carboxylic acid	>100	>100	>100	>100	>100	>100	>100	>100	>100
**6**	**6-Bromochromone-3-carbonitrile**	**5**	**5**	**50**	**20**	**20**	**20**	**10**	**>100**	**>100**
7	6-Bromo-3-formylchromone	50	50	50	50	50	20	20	50	50
8	3-Bromo-6-chlorochromone	50	50	100	50	50	20	20	20	20
9	Chromone	>100	>100	>100	>100	>100	>100	>100	>100	>100
10	Chromone-2-carboxylic acid	>100	>100	>100	>100	>100	>100	>100	>100	>100
11	Chromone-3-carboxylic acid	>100	>100	>100	>100	>100	>100	>100	>100	>100
**12**	**Chromone-3-carbonitrile**	**10**	**10**	**20**	**50**	**20**	**50**	**20**	**>100**	**>100**
13	6-Chloro-3-formylchromone	20	10	50	50	100	100	100	100	100
14	6-Chlorochromone	>100	>100	>100	>100	>100	>100	>100	>100	>100
15	6-Chlorochromone-2-carboxylic acid	>100	>100	>100	>100	>100	>100	>100	>100	>100
16	6-Chloro-7-methylchromone	>100	>100	>100	>100	>100	>100	>100	>100	>100
17	6,8-Dichlorochromone-3-carbonitrile	10	10	100	>100	>100	>100	>100	>100	>100
18	3-Formyl-6-isopropylchromone	50	50	100	100	50	50	50	50	50
19	3-Formyl-6-nitrochromone	20	20	50	50	20	20	20	>100	>100
20	3-Formyl-6-methylchromone	50	100	100	50	50	50	50	100	100
21	3-Formyl-6-methoxychromone	100	100	>100	>100	100	100	100	100	>100
22	6-Fluorochromone-2-carboxylic acid	>100	>100	>100	>100	>100	>100	>100	>100	>100
**23**	**6-Isopropylchromone-3-carbonitrile**	**10**	**10**	**20**	**10**	**10**	**10**	**10**	**>100**	**>100**
24	6-Methylchromone	>100	>100	>100	>100	>100	>100	>100	>100	>100
**25**	**6-Methylchromone-3-carbonitrile**	**10**	**10**	**20**	**10**	**20**	**50**	**20**	**>100**	**>100**
26	6-Methylchromone-2-carboxylic acid	>100	>100	>100	>100	>100	>100	>100	>100	>100
27	6-Nitrochromone	>100	>100	>100	>100	>100	>100	>100	>100	>100
28	Amphotericin B	1	1	0.5	0.5	5	5	2	0.5	0.5

^*a*^ The four selected compounds are indicated in bold font.

### Antibiofilm activities of chromones against *C. albicans*

**Fig 1:**
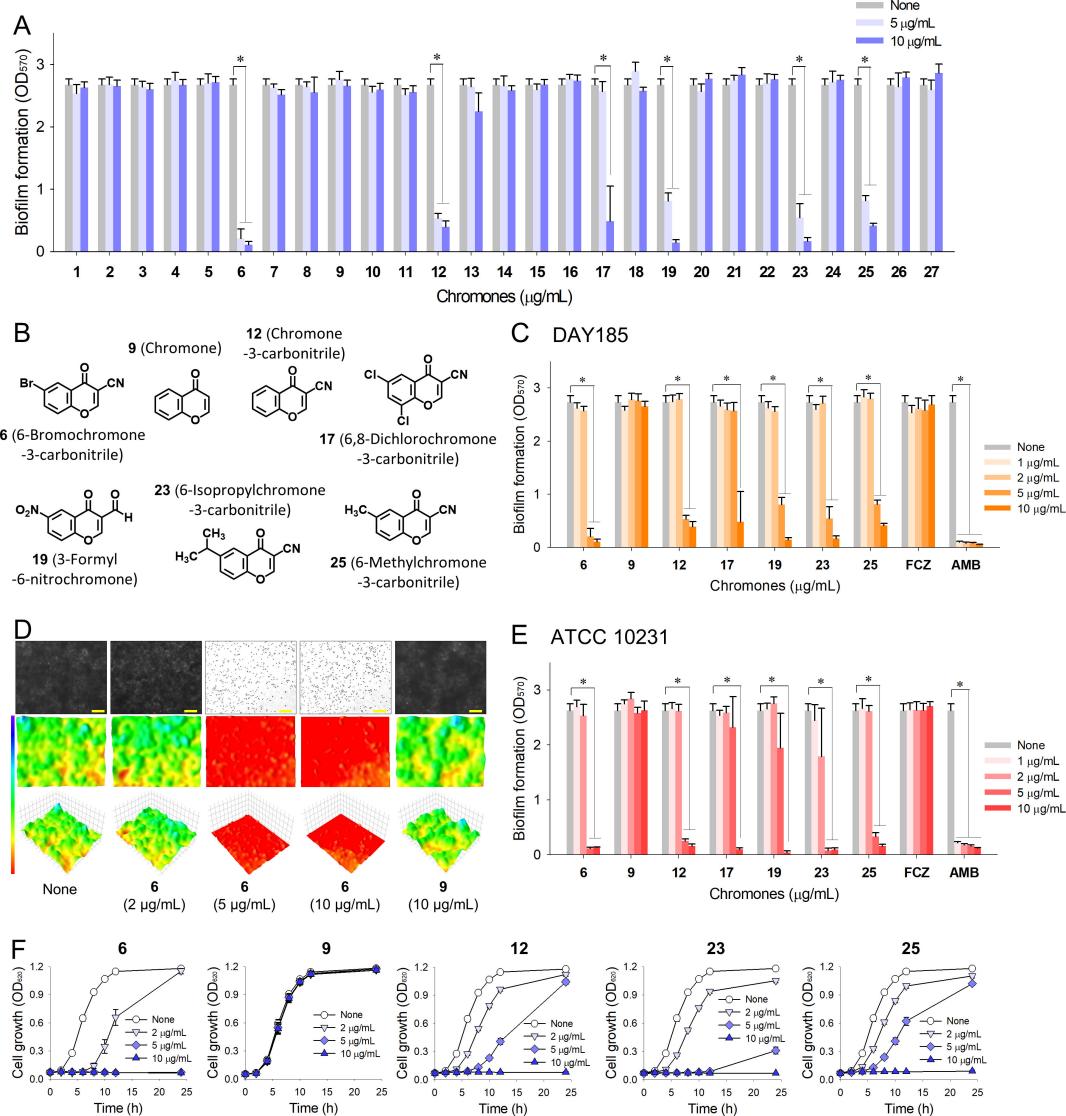
Antibiofilm activity of chromones. (**A**) Biofilm formation by *C. albicans* DAY185 in the presence of chromones at 5 or 10 µg/mL, (**B**) structures of representative chromones, (**C**) biofilm formation by *C. albicans* DAY185 in the presence of chromones, (**D**) color-coded 2D/3D images of *C. albicans* DAY185 biofilms in the presence of chromones, (**E**) biofilm formation by *C. albicans* ATCC10231 in the presence of chromones, and (**F**) planktonic cell growth of *C. albicans* DAY185 in the presence of chromones. Error bars indicate standard deviations. *P < 0.05 vs non-treated controls (None). Yellow scale bars represent 100 µm. None: non-treated control.

The antibiofilm activities of the 27 chromones at 5 or 10 µg/mL were initially examined against fluconazole-resistant *C. albicans* DAY185 on 96-well polystyrene plates, and several exhibited antibiofilm activity (Fig. 1A). As expected, fluconazole did not affect *C. albicans* biofilm formation, but amphotericin B dose-dependently inhibited it due to planktonic cell growth inhibition (Fig. 1C and E). Notably, 6,8-dichlorochromone-3-carbonitrile, 3-formyl-6-nitrochromone, and four chromone-3-carbonitriles, at 10 µg/mL inhibited *C. albicans* biofilm formation by >95% (Fig. 1A and B).

A more detailed biofilm study showed that six chromones mentioned above dose-dependently inhibited biofilm formation by fluconazole-resistant *C. albicans* DAY185 (Fig. 1C). Furthermore, four chromone-3-carbonitriles, namely, 6-bromochromone-3-carbonitrile, chromone-3-carbonitrile, 6-isopropylchromone-3-carbonitrile, and 6-methylchromone-3-carbonitrile, at 5 µg/mL inhibited *C. albicans* biofilm formation by >70% (Fig. 1C), whereas the backbone chromone (**9**) up to 100 µg/mL could not inhibit the biofilm formation. Microscopic observations confirmed *C. albicans* biofilm inhibition by 6-bromochromone-3-carbonitrile (Fig. 1D); for example, at 5 or 10 µg/mL, this derivative abolished biofilm formation, whereas chromone at 10 µg/mL had no effect. The antibiofilm activities of the six active chromones were also tested against a fluconazole-sensitive *C. albicans* ATCC 10231 strain, and four chromone-3-carbonitriles dose-dependently inhibited biofilm formation as expected (Fig. 1E).

In addition, the effects of four chromone-3-carbonitriles on planktonic cell growth were investigated. The MIC of 6-bromochromone-3-carbonitrile (the most active derivative) was 5 µg/mL (20 µM), whereas chromone (**9**) did not affect planktonic cell growth at concentrations ≤10 µg/mL (Fig. 1F). Four chromone-3-carbonitriles (**6**, **12**, **23**, and **25**) and the fluconazole-resistant *C. albicans* DAY185 strain were selected for further study based on their potent antifungal and antibiofilm activities; chromone was included for comparison purposes.

Furthermore, the ability of biofilm dispersal was evaluated against preformed *C. albicans* biofilms for 6 or 24 h. It appears that 6-bromochromone-3-carbonitrile, chromone, fluconazole, and amphotericin B up to 8× MIC could not eradicate the 24 h established biofilms (Fig. S2), confirming that biofilm eradication is more problematic than biofilm inhibition.

### The four chromone-3-carbonitriles inhibited hypha development and cell aggregation by *C. albicans*

**Fig 2:**
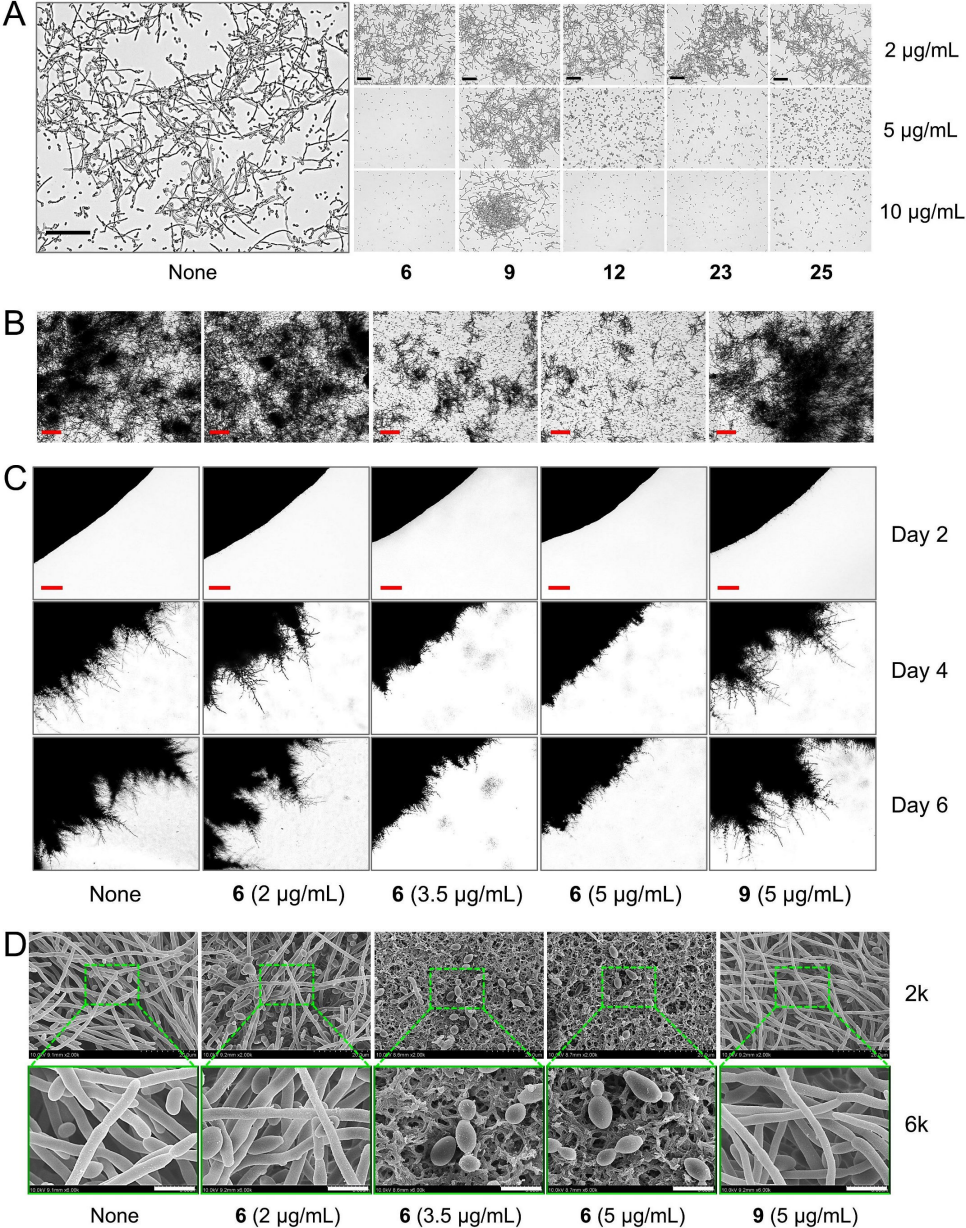
Inhibition of hyphal filamentation and aggregation by chromone-3-carbonitriles. (**A**) Hypha formation, (**B**) cell aggregation, (**C**) colony morphology, and (**D**) SEM images of *C. albicans*. 6, 9, 12, 23, and 25 represent 6-bromochromone-3-carbonitrile, chromone, chromone-3-carbonitrile, 6-isopropylchromone-3-carbonitrile, and 6-methylchromone-3-carbonitrile, respectively. Black, red, and white bars represent 100, 200, and 5 µm, respectively.

Hyphal transition and cell aggregation are prerequisites for *C. albicans* biofilm formation, and thus, we subjected the four chromone-3-carbonitriles to hypha formation, cell aggregation, and protrusion assays using *C. albicans* DAY185. In untreated controls, hypha and yeast cells were simultaneously observed in potato dextrose broth (PDB) medium (Fig. 2A). The four chromone-3-carbonitriles inhibited hypha formation at 5 and 10 µg/mL. Also, 6-bromochromone-3-carbonitrile dose-dependently inhibited cell aggregation in PDB containing 10% bovine serum (Fig. 2B), and when treated at 3.5 or 5 µg/mL for 6 days, suppressed hyphal protrusions from colonies of untreated *C. albicans* incubated for 4 days (Fig. 2C). SEM analysis confirmed that biofilms of non-treated controls on nylon membranes were composed of hyphae and few yeast cells and that 6-bromochromone-3-carbonitrile at 3.5 or 5 µg/mL reduced hyphal lengths and *C. albicans* adhesion to these membranes. These observations suggested that chromone-3-carbonitriles inhibit yeast-to-hyphal transition and cell aggregation and thus inhibit *C. albicans* biofilm development and virulence (Fig. 2D).

### Gene expressional changes induced by chromone-3-carbonitrile in *C. albicans*

**Fig 3:**
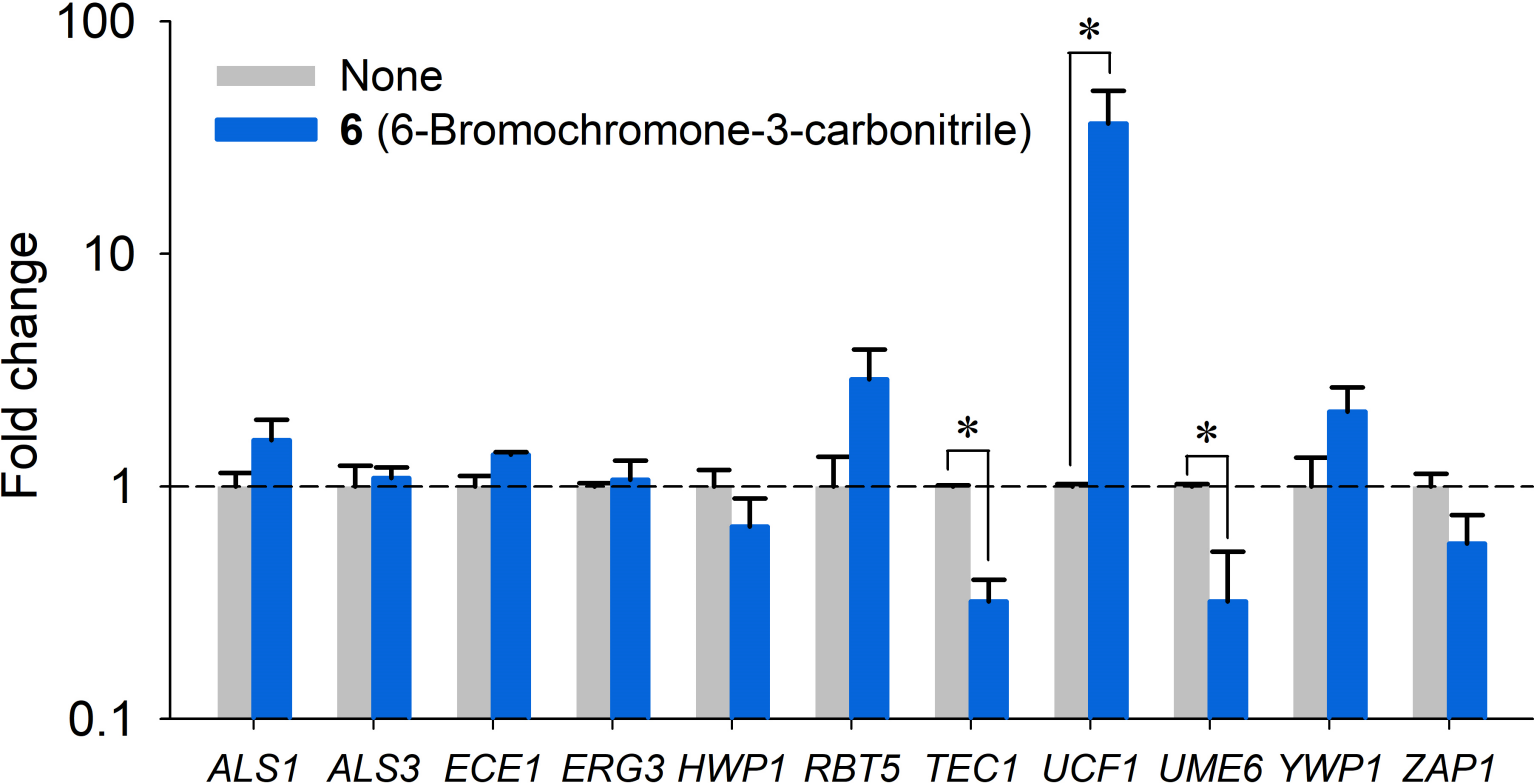
Effects of 6-bromochromone-3-carbonitrile on gene expressions in *C. albicans*. Relative transcriptional profiles of biofilm-related genes in *C. albicans*. Cells were treated with 6-bromochromone-3-carbonitrile at 3.5 µg/mL for 4 h without shaking. Fold changes indicate transcriptional differences observed in treated vs untreated (None) cells as determined by qRT-PCR. *RDN18* was used as the housekeeping gene. *P < 0.05 vs non-treated controls.

qRT-PCR was used to explore the expressions of 11 biofilm- and hypha-related genes in *C. albicans* DAY185 after treating *C. albicans* with 6-bromochromone-3-carbonitrile at 3.5 µg/mL (14 µM) for 4 h. This derivative downregulated the expressions of *TEC1* (a biofilm-related gene) by 3.1-fold and *UME6* (a filament-specific regulator) by 3.1-fold but upregulated *UCF1* (a hypha regulator) by 36-fold but did not alter the expressions of the other genes tested (Fig. 3). qRT-PCR results showed chromone-3-carbonitrile downregulated the expressions of these hypha- and biofilm-related genes, supporting its inhibition of hypha and biofilm development.

### Chemical toxicities of chromones in the nematode and plant models

**Fig 4:**
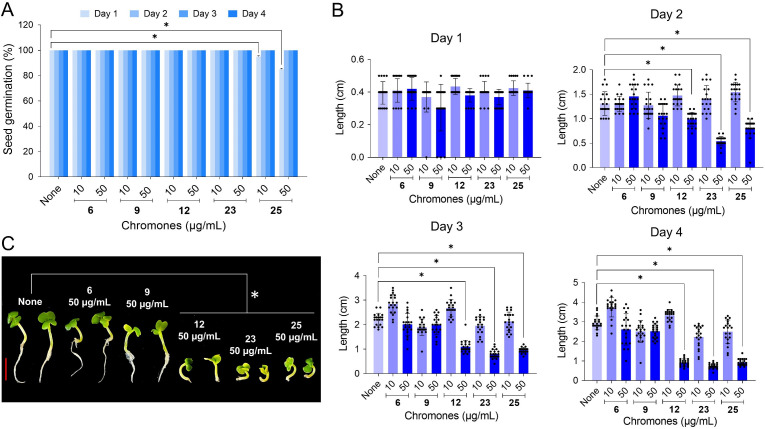
Chemical toxicities of chromones as determined using the plant germination. (**A**) *B. rapa* seed germination assays were performed using Murashige and Skoog agar medium supplemented with or without chromones at 25°C. (**B and C**) Plant total lengths were analyzed on 1, 2, 3, and 4 days. Red scale bar indicates 1 cm. 6, 9, 12, 23, and 25 represent 6-bromochromone-3-carbonitrile, chromone, chromone-3-carbonitrile, 6-isopropylchromone-3-carbonitrile, and 6-methylchromone-3-carbonitrile, respectively.

The toxicities of four chromone-3-carbonitriles were examined using a *Caenorhabditis elegans* model and a *Brassica rapa* seed germination model since *in vivo* nematode survival model has been widely used to explore chemical toxicity and we wanted to study the environmental effect of chromone derivatives on plants. Seed germination rate was unaffected by 6-bromochromone-3-carbonitrile (**6**), chromone-3-carbonitrile (**12**), or 6-isopropylchromone-3-carbonitrile (**23**) at 10 or 50 µg/mL, but 6-methylchromone-3-carbonitrile (**25**) caused a slight reduction (Fig. 4A). Plant heights were similar after 6-bromochromone-3-carbonitrile (**6**) or chromone (**9**) treatment for 4 days, whereas chromone-3-carbonitrile (**12**), 6-isopropylchromone-3-carbonitrile (**23**), and 6-methylchromone-3-carbonitrile (**25**) at 10 or 50 µg/mL inhibited plant growth (Fig. 4B and C). These results suggested that 6-bromochromone-3-carbonitrile (**6**) was the least phytotoxic.

**Fig 5:**
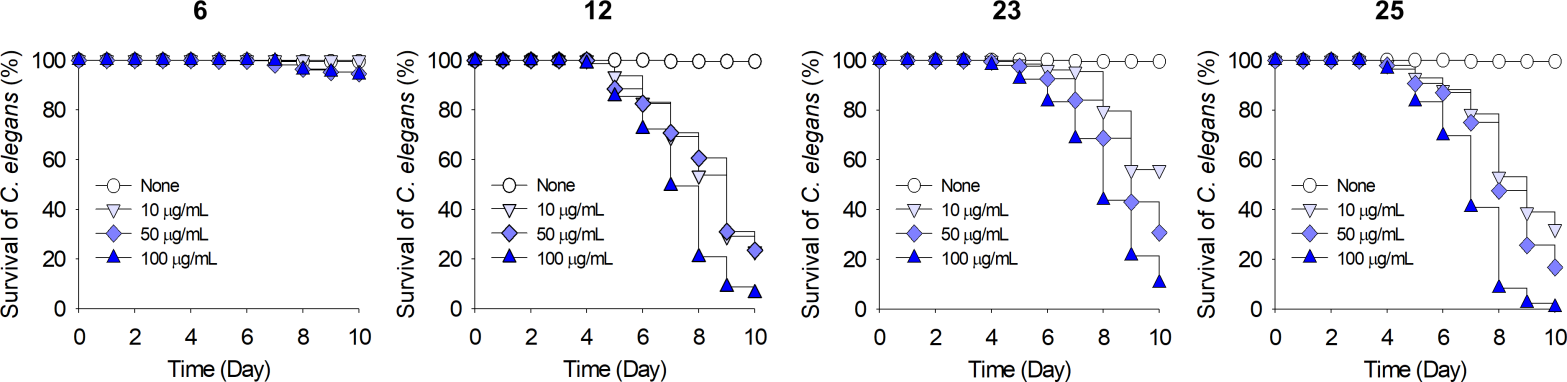
Chemical toxicities of chromones on nematode models. *C. elegans* survival was assessed in the presence or absence of chromones for 10 days. 6, 9, 12, 23, and 25 represent 6-bromochromone-3-carbonitrile, chromone, chromone-3-carbonitrile, 6-isopropylchromone-3-carbonitrile, and 6-methylchromone-3-carbonitrile, respectively.

The toxicities of chromone-3-carbonitriles were also investigated using nematode *C. elegans*. As was observed for *B. rapa* seed germination, 6-bromochromone-3-carbonitrile was less toxic to *C. elegans* (Fig. 5). More specifically, most nematodes survived after treatment with 6-bromochromone-3-carbonitrile at 100 µg/mL for 10 days, whereas the majority died after treatment with the other chromone-3-carbonitriles at 10–100 µg/mL. These results show that 6-bromochromone-3-carbonitrile, in its active antifungal and antibiofilm range (3.5–10 µg/mL), does not affect *B. rapa* growth and/or *C. elegans* survival.

### ADME profiling of chromone-3-carbonitriles

ADME profiles of chromone and the four chromone-3-carbonitriles were also evaluated. All five chromones complied with Lipinski’s rule of five. Furthermore, all four had bearable skin and brain barrier permeabilities and human intestinal adsorptions, were non-carcinogenic to mice, and did not exhibit acute fish toxicity. Full ADME profiles are presented in Table S2.

## DISCUSSION

The present study shows four chromone-3-carbonitriles are potent anti-*Candida* agents with anti-hyphal and antibiofilm activities. Their action mechanism has been partially revealed and non-toxicological characteristics have been demonstrated.

The antifungal activities of chromone and some chromone derivatives, including sulfonamide-, hydroxy-pyrazolyl-, azo-, oxepino-, methoxy-fluorophenyl-, and methoxy-fluorophenyl-chromones and chromanone A, have been reported (7, 9 – 13), but this is the first report to be issued on the antifungal activities of chromone-3-carbonitriles (Table 1; Fig. 1). While chromone itself showed weak antifungal activity, several compounds with a 3-carbonitrile motif have been previously reported to possess antifungal activity. For example, tetrahydroquinoline-3-carbonitriles by the inhibition of chitin synthase activity (14), benzochromene-3-carbonitriles possibly by binding cytochrome CYP51 site (15), benzoxazole-4-carbonitriles as a glucan synthesis inhibitor (16), pyridine-3-carbonitrile (17), and 2-substituted-phenyl-1H-benzimidazole-5-carbonitriles (18) exhibited moderate antifungal activity, which showed the carbonitrile motif can confer antifungal activity. Hence, it is possible that chromone-3-carbonitriles act on cell wall components such as chitin, glucans, or membrane proteins.

Appreciating the importance of strain specificity, the efficacies of antifungal activity of chromone derivatives were accessed on nine *Candida* strains, including *C. albicans*, *C. glabrata*, *C. parapsilosis*, and *C. auris* (Table 1). MICs of 6-bromochromone-3-carbonitrile (**6**), chromone-3-carbonitrile (**12**), 6-isopropylchromone-3-carbonitrile (**23**), and 6-methylchromone-3-carbonitrile (**25**) were 5–50 µg/mL on six strains of *C. albicans*, *C. glabrata*, and *C. parapsilosis* but their MICs on *C. auris* strains were ≥100 µg/mL. Hence, the antifungal efficacies of four chromone-3-carbonitriles are *Candida*-specific, and *C. auris* strains are less susceptible to the chromone-3-carbonitrile. Previously, chromone derivatives such as chromanone A (19), 3-((1-benzyl-1H-1,2,3-triazol-4-yl)methoxy)-2-(4-fluorophenyl)-4H-chromen-4-ones (10), and 2,6-dimethyl-5-methoxyl-7-hydroxylchromone (20) demonstrated antibacterial and antifungal activities, which partially agree with current findings.

In addition to antifungal activity, antibiofilm and antivirulence strategies are alternative and supportive means to control recalcitrant *Candida* infections. Previously, chromone derivatives such as 8-methoxy-3-methyl-4-oxo-4H-chromene-2-carbaldehyde (12) and chromone 5-maleimide substitution compounds (21) inhibited biofilm formation of *C. albicans* and *Staphylococcus aureus* by inhibiting hyphal pseudomycelium formation and cell adherence, respectively. The hyphal form of *C. albicans* is a prerequisite for pathogenicity and biofilm formation, and the regulatory mechanism responsible for hypha and biofilm development has been widely reported (22, 23). 6-bromochromone-3-carbonitrile inhibited hyphal formation and cell aggregation of *C. albicans* (Fig. 2) and it regulated the expressions of hypha-forming and biofilm-related genes (*TEC1*, *UME6*, and *UCF1*) in *C. albicans* (Fig. 3). *TEC1* is one of the six regulatory genes that control cell morphology and biofilm formation (23), whereas *UME6* is a key regulator of hyphal extension and biofilm formation (24, 25). Previously, *UCF1* expression was upregulated by antibiofilm agents such as nepodin (26) and medium-chain fatty acids (27) in *C. albicans*. Notably, the observed transcriptional changes induced by 6-bromochromone-3-carbonitrile concur with these previous findings (Fig. 3).

Four chromone-3-carbonitriles had no phytotoxic effect, did not affect *C. elegans* survival in its effective antifungal and antibiofilm range (Fig. 4 and 5), and exhibited drug-like ADME properties (Table S2). Further *in vivo* experiments are required to investigate the effectiveness of chromone-3-carbonitriles as anti-*Candida* agents. Overall, this study shows chromone-3-carbonitriles could offer a potential means of controlling *Candida* infections.

## MATERIALS AND METHODS

### *Candida* strains, reagents, and MICs

Two *C. albicans* strains, DAY185 (fluconazole-resistant) and ATCC 10231 (fluconazole-sensitive), were acquired from the Korean Culture Center for Microorganisms (KCCM) and the American Type Culture Collection (ATCC), respectively. *C. albicans* strains were cultivated in solid potato dextrose agar (PDA) for colony formation by streaking from glycerol stock or liquid PDB for all other experiments. *Candida glabrata* ATCC 2001, *Candida glabrata* KCCM 50701, *Candida parapsilosis* ATCC 7330, *Candida parapsilosis* ATCC 50030, and *Candida parapsilosis* ATCC 22019 were obtained from the ATCC or the KCCM and cultured in solid PDA for colony formation or liquid yeast malt medium supplemented with 2% glucose for MIC determination. Two *Candida auris* strains, KCTC17809 and KCTC17810, were obtained from the Korean Collection for Type Culture (KCTC) and cultivated in solid PDA for colony formation or liquid tryptic soy broth medium for MIC determination and biofilm formation (26). Four clinical isolates [three *Candida albicans* strains (KCCM 12552, KCCM 12555, and KCCM 12556) and *Candida tropicalis* (KCCM 5127)] were obtained from the KCCM and cultured in solid PDA for colony formation or liquid PDB medium for MIC determination. For all strains, cells streaked on PDA plates were incubated for 48 h at 37°C and a single colony was then selected and inoculated into 25 mL of appropriate liquid medium in 250 mL flat-bottomed flasks and cultured overnight at 37°C. The 27 chromones investigated were as follows: 2-amino-3-formylchromone, 2-amino-6-chloro-3-formylchromone, 3-bromochromone, 6-bromo-4H-chromen-4-one, 6-bromochromone-2-carboxylic acid, 6-bromochromone-3-carbonitrile, 6-bromo-3-formylchromone, 3-bromo-6-chlorochromone, chromone, chromone-2-carboxylic acid, chromone-3-carboxylic acid, chromone-3-carbonitrile, 6-chloro-3-formylchromone, 6-chlorochromone, 6-chlorochromone-2-carboxylic acid, 6-chloro-7-methylchromone, 6,8-dichlorochromone-3-carbonitrile, 3-formyl-6-isopropylchromone, 3-formyl-6-nitrochromone, 3-formyl-6-methylchromone, 3-formyl-6-methoxychromone, 6-fluorochromone-2-carboxylic acid, 6-isopropylchromone-3-carbonitrile, 6-methylchromone, 6-methylchromone-3-carbonitrile, 6-methylchromone-2-carboxylic acid, and 6-nitrochromone (Table 1). Chromones, fluconazole, and amphotericin B were purchased from Sigma-Aldrich (St. Louis, USA) or Combi-blocks (San Diego, USA) and dissolved in dimethyl sulfoxide (DMSO). DMSO concentrations in cultures did not exceed 0.1% (vol/vol). Planktonic cell growths were assessed using a spectrophotometer (Multiskan SkyHigh microplate reader; Thermo Fisher Scientific, Waltham, MA, USA) at 620 nm after cultivation for 24 h at 37°C. For MICs, Clinical Laboratory Standards Institute broth dilution method was used with a slight modification. *Candida* strains were cultured overnight in 25 mL appropriate liquid medium in 250 mL flat-bottomed flasks, then diluted 1:50 (~ 10^5^ CFU/mL) in the liquid medium, dispensed into each well of a 96-well plate having different concentrations (wt/vol) of the chromone derivatives, and incubated for 24 h at 37°C. MIC was decided as the lowest concentration in which planktonic cell growth was not detectable (25, 28).

### Microtiter plate biofilm assays

*C. albicans* biofilms were formed on 96-well polystyrene plates, as previously reported (29). Overnight cultures of *C. albicans* cells were sub-inoculated into fresh PDB at a turbidity of 0.1 (~ 10^5^ CFU/mL) at 600 nm and incubated with or without different concentrations of the 27 chromone derivatives for 24 h without agitation at 37°C. The spent media were then discarded, and plates were washed three times with clean water to remove planktonic cells. Biofilm cells were then stained with crystal violet (0.1% wt/vol, Sigma-Aldrich, St. Louis, USA) for 20 min, washed three times with clean water, and then the crystal violet was extracted using 95% ethanol. Absorbances of crystal violet extracts were measured using a Multiskan SkyHigh microplate reader at 570 nm. The results are expressed as the averages of at least six replicates.

Biofilm dispersal assay was performed with established biofilms of *C. albicans*. After biofilm formation for 6 or 24 h, chromone compounds and standard drugs up to 16× MIC were added. After another 24 h incubation, the biofilm formation was measured as above.

### Microscopic observations of *C. albicans* biofilm formation

*C. albicans* DAY185 biofilms were developed over 24 h at 37°C, as described above, and free-floating cells and spent medium were removed by gentle washing with clean water three times. Biofilms were visualized by live imaging microscopy using the iRiS Digital Cell Imaging System (Logos BioSystems), and biofilm images were generated as color-coded 2D and 3D images using ImageJ (https://imagej.nih.gov/ij) (25).

### *C. albicans* hypha development and cell aggregation

*C. albicans* DAY185 cells were sub-inoculated into 1 mL of PDB medium at a cell density of 10^5^ CFU/mL in 1.7 mL tubes with or without the chromone derivatives (2, 5, or 10 µg/mL) and incubated without agitation at 37°C for 24 h (25). Cell cultures (1 mL) were gently vortexed for a few seconds, transferred to glass-bottomed dishes, and observed in a bright field under an optical microscope, iRiS Digital Cell Imaging System (Logos BioSystems, Anyang, Republic of Korea) at 4× and 10× (27). To investigate *C. albicans* colony morphology on solid PDA medium, glycerol stocks were streaked on PDA plates containing chromone derivatives (2, 3.5, or 5 µg/mL) and incubated for 6 days at 37°C. Colony morphologies were observed under an optical microscope (iRiS Digital Cell Imaging System). At least four independent experiments were conducted.

### Examination of hyphae formation by SEM

SEM was used to analyze hypha formation, as previously reported (30). A sterile nylon membrane (0.2 µm pore size, Whatman, Maidstone, UK) was cut into small pieces (~ 0.4 × 0.4 cm), and single pieces were placed in each well of 96-well plates having 300 µL of cell culture of turbidity 0.1 at 600 nm. Cells were then incubated with or without each of the chromone derivatives for 24 h at 37°C without shaking. Biofilm cells on nylon membranes were then fixed with a mixture of glutaraldehyde (2.5%) and formaldehyde (2%) for 30 h at 4°C, post-fixed in 1% osmium tetroxide (OsO_4_), and dehydrated by incubation in an ethanol series (50%, 70%, 80%, 90%, 95%, and 100%). After critical-point drying, biofilm cells on nylon membranes were visualized under a field emission scanning electron microscope (S-4800, Hitachi, Japan) at a voltage of 10 kV and 500–5000 × magnifications.

### RNA and quantitative real-time PCR

For transcriptomic analysis, *C. albicans* DAY185 was inoculated into 25 mL of fresh PDB in 250 mL flat-bottomed flasks at an OD_600_ of ~0.1 and incubated for 3 h at 37°C with shaking at 250 rpm in the presence or absence of 6-bromochromone-3-carbonitrile (3.5 µg/mL). RNase inhibitor (RNAlater; Ambion, TX, USA) was then added to prevent RNA degradation and mixed gently. Total RNA was isolated from *C. albicans* and purified using an RNeasy mini Kit (Qiagen, Hilden, Germany), as previously described (31). Isolated and purified RNA was quantified using a NanoVue UV-Vis spectrophotometer (GE Healthcare, Chicago, USA). qRT-PCR was performed using gene-specific primers to measure the transcription quantities of hypha- and biofilm-related genes [*ALS1, ALS3, ECE1, ERG3, HWP1* (also called *ECE2*)*, RBT5, TEC1, UCF1, UME6, YWP1,* and *ZAP1*]. *RDN18* was used as the housekeeping control (Table S3). qRT-PCR was conducted using SYBR Green real-time PCR master mix (Thermo Fisher Scientific, Waltham, USA) and a StepOne Real-Time PCR System (Applied Biosystems, Foster City, USA) (26).

### Chemical toxicity assays using the seed germination model

The germination and plant growth of Chinese cabbage (*Brassica rapa*) were used to investigate the toxicities of four selected chromones, as previously reported (25). Briefly, seeds of *B. rapa* were washed three times with sterile water, soaked in sterile distilled water for 16 h, and seed surfaces were sterilized by incubating seeds sequentially in 95% ethanol and 3% sodium hypochlorite for 20 min at 25°C and washed with sterile water three times. Seeds were then seeded on soft agar Murashige and Skoog plates (0.7% agar and 0.86 g/L Murashige and Skoog) with or without chromone derivatives (10 or 50 µg/mL) and incubated at room temperature (24°C) for 4 days. Seed germination rates and total plant lengths were measured daily. Four independent experiments were performed at each concentration.

### Chemical toxicity assays using the nematode model

To explore the chemical toxicity of four selected chromones, we used *C. elegans* strain *fer-15(b26)* and *fem-1(hc17*), as previously described (27). Briefly, synchronized young adult nematodes were cleaned twice with M9 buffer (3 g/L KH_2_PO_4_, 6 g/L Na_2_HPO_4_, 5 g/L NaCl, 1 mM MgSO_4_), and ~30 worms were dispensed into each well of 96-well plates containing M9 buffer (200 µL) and a chromone derivative (10, 50, or 100 µg/mL). Plates were then set for 10 days at 25°C without agitation. Four independent experiments were performed in triplicate. Results are exhibited as percentages of live worms, as decided by responses to plate tapping and LED lights for 20 to 30 s using an iRiS Digital Cell Imaging System (Logos BioSystems).

### Simulation of absorption, distribution, metabolic, and excretion properties

The drug-like properties of the four selected chromones were evaluated using ADME software (32). The online web servers PreADMET (https://preadmet.qsarhub.com/), Molinspiration (https://www.molinspiration.com/), and GUSAR (http://www.way2drug.com/gusar/) were accessed on 5 February 5, 2023. According to Lipinski’s rule of five, an orally active drug should have a molecular weight of ≤500 g/mol, a Log *P* of ≤5, ≤5 hydrogen bond-donating atoms, ≤10 hydrogen-bond accepting atoms, and an octanol-water partition coefficient of ≤140 Å^2^ (33).

### Statistical analysis

The analysis was conducted by one-way ANOVA followed by Dunnett’s test in SPSS version 23 (SPSS Inc., Chicago, IL, USA). *P* values < 0.05 were deemed significant. Asterisks designate significant differences between untreated and chemical-treated samples, and results are expressed as means ± standard deviations. Sample replication numbers are provided above.

## SUPPLEMENTAL MATERIAL

Supplemental file 1 spectrum.01737-23-s0001.pdf Tables S1 to S3 and Fig. S1 and S2.

ASM does not own the copyrights to Supplemental Material that may be linked to, or accessed through, an article. The authors have granted ASM a non-exclusive, world-wide license to publish the Supplemental Material files. Please contact the corresponding author directly for reuse.
