# Supplementary material for: Antifungal and antibiofilm activities of chromones against nine Candida species
Source: Microbiol Spectr. 2023 Oct 24;11(6):e01737-23. doi: 10.1128/spectrum.01737-23 (PMC10714962; doi:10.1128/spectrum.01737-23)
Supplement: Supplemental file 1 — Tables S1 to S3 and Fig. S1 and S2. [file spectrum.01737-23-s0001.pdf]

Antifungal and antibiofilm activities of chromones against nine *Candida* species

Jin-Hyung Lee[a], Yong-Guy Kim[a], Yeseul Kim[a], and Jintae Lee*

School of Chemical Engineering, Yeungnam University, Gyeongsan, Republic of Korea

[a]Jin-Hyung Lee and Yong-Guy Kim and contributed equally to this article. Author order was determined alphabetically.

*Address correspondence to

Jintae Lee, jtlee@ynu.ac.kr

Tel.: +82-53-810-2533, Fax: +82-53-810-4631

Running title: Antifungal activity of chromones.

Table S1. Antifungal activity of chromones against clinical isolates. #6 and #9 indicate 6-bromochromone-3-carbonitrile and chromone.

**ID (KCCM)**	**Strain name**	**Origin**	**MIC**		
			**Amphotericin B**	**#6**	**#9**
**12552**	*Candida albicans*	National Hospital Kyoto	0.5 μg/mL	5 μg/mL	>100 μg/mL
**12555**	*Candida albicans*	Isolated from the vaginal tracts	0.2 μg/mL	2 μg/mL	>100 μg/mL
**12556**	*Candida albicans*	Division of mycotic diseases	0.5 μg/mL	5 μg/mL	>100 μg/mL
**51287**	*Candida tropicalis*	Rumen fluid, Gyeongsang National University	0.5 μg/mL	5 μg/mL	>100 μg/mL

Table S2. ADME profile of five chromones (6-bromochromone-3-carbonitrile **(6)**, chromone **(9)**, chromone-3-carbonitrile **(12)**, 6-isopropylchromone-3-carbonitrile **(23)**, and 6-methylchromone-3-carbonitrile **(25)**). The profile is an amalgamation of the ADME properties of five chromones from the online webservers, viz. PreADMET, Molinspiration and GUSAR.

**Property**	**6**	**9**	**12**	**23**	**25**
Lipinski rule of five	Suitable	Suitable	Suitable	Suitable	Suitable
Lipinski rule of five violations	0	0	0	0	0
In vivo blood brain barrier penetration	1.80458	1.66208	1.17031	2.09915	1.58509
In vitro Caco2 cell permeability	21.1163	46.997	17.1627	4.95789	19.2641
Human intestinal absorption (%)	98.071571	100	97.058098	97.971776	97.456592
In vitro plasma protein binding (%)	99.074407	73.152102	92.616426	100	94.003753
In vivo skin permeability	-3.10383	-2.24556	-3.16961	-2.99313	-3.02562
Mouse carcinogenicity	Positive	Positive	Negative	Positive	Negative
Rat carcinogenicity	Positive	Positive	Positive	Positive	Positive
In vitro hERG inhibition	low_risk	medium_risk	medium_risk	medium_risk	medium_risk
miLogP	2.29	1.82	1.5	2.99	1.93
TPSA	54	30.21	54	54	54
Mol volume	163.33	128.59	145.45	195.4	162.01
GPCR ligand	-1.02	-1.09	-0.92	-0.56	-0.86
Ion channel modulator	-0.96	-0.73	-0.86	-0.64	-0.91
Kinase inhibitor	-0.78	-1.39	-0.82	-0.56	-0.78
Nuclear receptor ligand	-0.98	-1.44	-0.84	-0.38	-0.75
Protease inhibitor	-1.47	-1.59	-1.37	-0.95	-1.31
Enzyme inhibitor	-0.43	-0.42	-0.34	-0.14	-0.36
Rat IP LD50 classification	Class 5 out of AD	Class 4 in AD	Class 4 in AD	Class 4 out of AD	Class 4 out of AD
Rat IV LD50 classification	Class 4 in AD	Class 3 in AD	Class 4 in AD	Class 3 in AD	Class 3 in AD
Rat oral LD50 classification	Class 4 in AD	Class 4 in AD	Class 4 in AD	Class 4 in AD	Class 4 in AD
Rat SC LD50 classification	Class 5 in AD	Class 5 in AD	Class 5 in AD	Class 4 in AD	Class 5 in AD

Table S3. Primer sequences used for qRT-PCR.

Gene	Function	Primer
ALS1	Cell-surface adhesion, adhesion/invasion, virulence	Forward 5'-AGC TGT TGC CAG TGC TTC-3'<br>Reverse 5'-AAT GTG TTG GTT GAA GGT GAG-3'
ALS3	Cell wall adhesion; epithelial adhesion	Forward 5'-CAA CAT CAA CCA ACC AAT CTC-3'<br>Reverse 5'-TGA ATA ACA GAA CCA GAT CCG-3'
ECE1	Candidalysin, cytolytic peptide toxin essential for mucosal infection; hypha-specific protein	Forward 5'-CCA GAA ATT GTT GCT CGT GTT GCC A-3'<br>Reverse 5'-TCC AGG ACG CCA TCA AAA ACG TTA G-3'
ERG3	C-5 sterol desaturase; hyphal growth and virulence	Forward 5'-CAT AAA CCT CAT CAC AAG TGG ATT G-3’<br>Reverse 5'-AAA GAT TGG AAG AACCCATCAACT-3’
HWP1	Hyphal cell wall protein; biofilm	Forward 5'-TTG TTT GCG TCA TCA AGA CTT TG-3'<br>Reverse 5'-GTC TTC ATC AGC AGT AAC ACA ACC A-3'
RBT5	GPI-linked cell wall protein; biofilm	Forward 5'-CTG CTG AAA GTT CTG CAC CA - 3'<br>Reverse 5'-GCT TCA ACG GAA ACA GAA GC - 3'
TEC1	TEA/ATTS transcription factor	Forward 5'-GGC CAT GAG AGA ACA ATA TA-3'<br>Reverse 5'-GTC TTT CCA TTT CTA AAT CAC-3'
UCF1	Upregulated by cAMP in filamentous growth; biofilm	Forward 5'-ATG GCG GGA AAG AAA AAG TC-3'<br>Reverse 5'-CCC AAG TTT CAT CAC GAA CA-3'
UME6	Zn(II)2Cys6 transcription factor; hyphal extension, virulence, adherence	Forward 5'-AGC ACC AAA TTC GCC TTA TG-3'<br>Reverse 5'-AGG TTG AGC TTG CTG CAG TT-3'
YWP1	Secreted yeast wall protein; biofilm dispersal	Forward 5'-GTT CCA TTT TTC CAA GTT CAT TTA G-3'<br>Reverse 5'-TCA AGA GTA GAA CCT TCA AGA GCA G-3'
ZAP1	Zinc-regulated transcription factor	Forward 5'-CGA CTA CAA ACC ACC AGC TTC ATC-3'<br>Reverse 5'-CCC CTG TTG CTC ATG TTT TGT T-3'
RDN18	18S ribosomal RNA; Housekeeping	Forward 5'-AGA AAC GGC TAC CAC ATC CCA-3'<br>Reverse 5'-CGA ATG GGC CCT GTA TCG T-3'

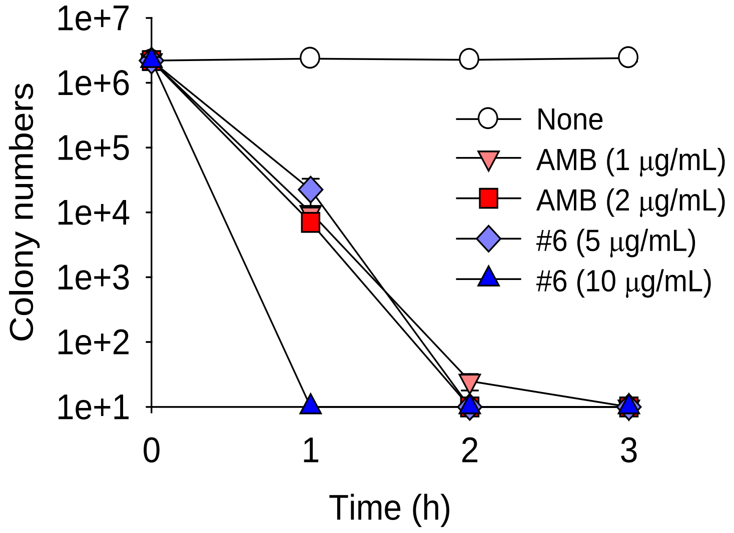

Fig. S1. Fungicidal activity of chromones against *C. albicans* DAY185. AMB and #6 indicate amphotericin B and 6-bromochromone-3-carbonitrile.

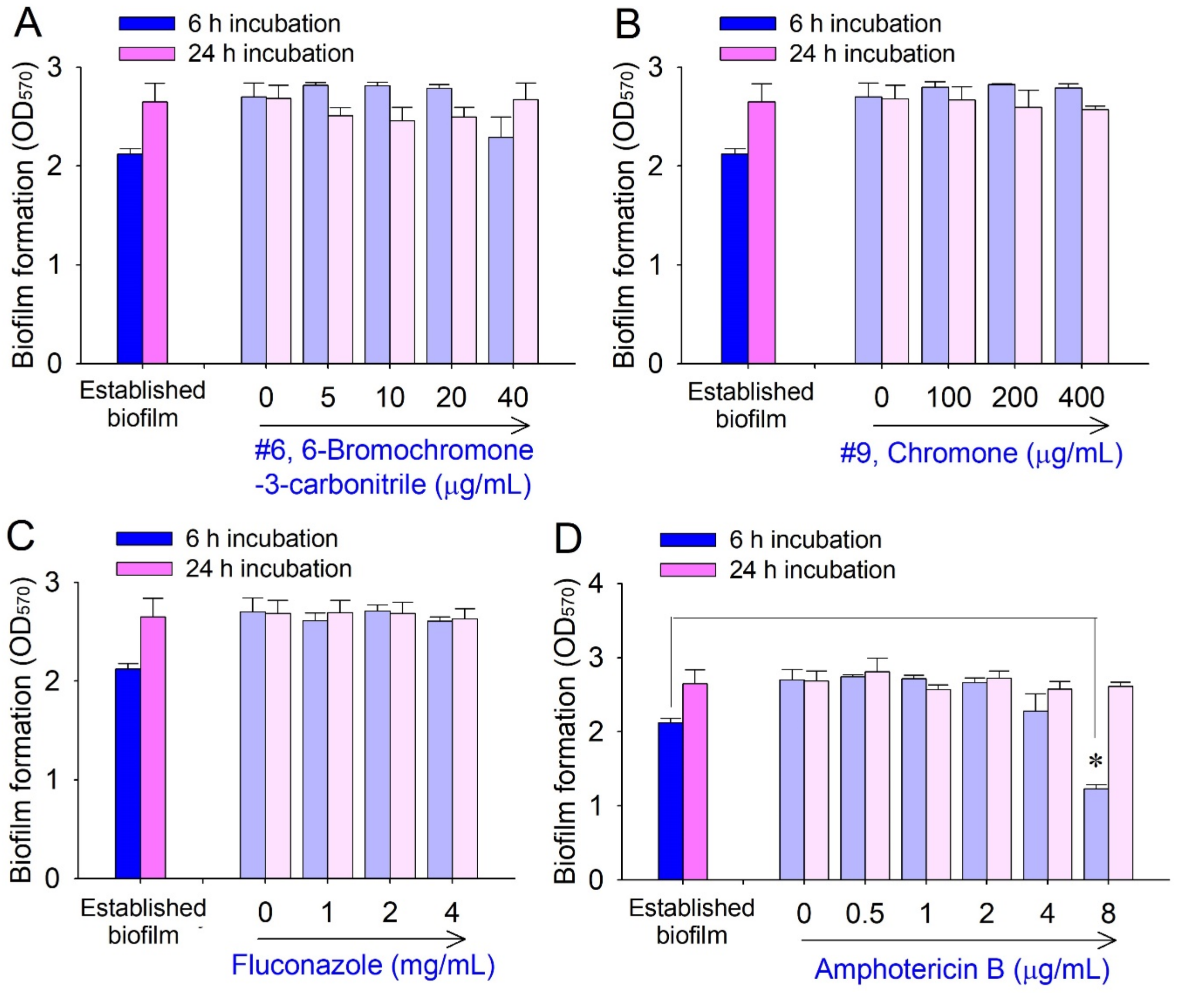

Fig. S2. Effects of chromones on biofilm dispersal. Biofilm of *C. albicans* DAY185 was developed by incubation for 6 h or 24 h. After biofilm formation, culture medium was discarded and fresh PDB medium having (A) 6-bromochromone-3-carbonitrile, (B) chromone, (C) fluconazole, or (D) amphotericin B was added and incubated for additional 24 h.
